# l-Asparaginase Immobilized on Nanographene Oxide as an Efficient Nanobiocatalytic Tool for Asparagine Depletion in Leukemia Cells

**DOI:** 10.1021/acs.bioconjchem.4c00518

**Published:** 2025-01-14

**Authors:** Paulina Erwardt, Bartosz Szymczak, Marek Wiśniewski, Bartosz Maciejewski, Michał Świdziński, Janusz Strzelecki, Wiesław Nowak, Katarzyna Roszek

**Affiliations:** † Department of Materials Chemistry, Adsorption and Catalysis, Faculty of Chemistry, Nicolaus Copernicus University in Torun, ul. Gagarina 7, 87-100 Torun, Poland; ‡ Department of Biochemistry, Faculty of Biological and Veterinary Sciences, Nicolaus Copernicus University in Torun, ul. Lwowska 1, 87-100 Torun, Poland; § Department of Immunology, Faculty of Biological and Veterinary Sciences, Nicolaus Copernicus University in Torun, ul. Lwowska 1, 87-100 Torun, Poland; ∥ Department of Cellular and Molecular Biology, Faculty of Biological and Veterinary Sciences, Nicolaus Copernicus University in Torun, ul. Lwowska 1, 87-100 Torun, Poland; ⊥ Department of Biophysics, Institute of Physics, Faculty of Physics, Astronomy and Informatics, Nicolaus Copernicus University in Torun, ul. Grudziądzka 5, 87-100 Torun, Poland

## Abstract

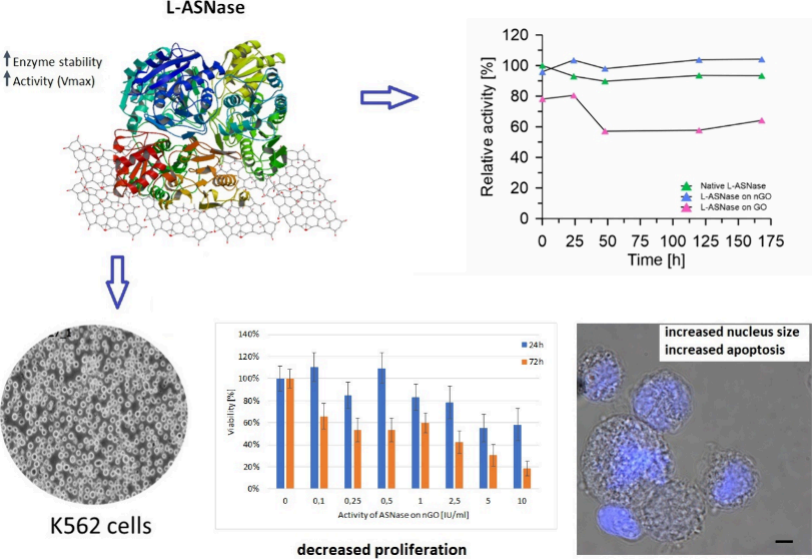

l-Asparaginase (l-ASNase) catalyzes the hydrolysis of l-asparagine, leading to its depletion and subsequent effects on the cellular proliferation and survival. In contrast to normal cells, malignant cells that lack asparagine synthase are extremely susceptible to asparagine deficiency. l-ASNase has been successfully employed in treating pediatric leukemias and non-Hodgkin lymphomas; however, its usage in adult patients and other types of cancer is limited due to significant side effects and drug resistance. Recent research has explored alternative formulations and delivery methods to enhance its efficacy and minimize adverse effects. One promising approach involves the immobilization of l-ASNase onto nanostructured materials, offering improved enzymatic activity and biocompatibility of the support. We harnessed an *E. coli*l-ASNase type II preparation to develop a novel strategy of enzyme immobilization on graphene oxide (GO)-based support. We compared GO and nanographene oxide (nGO) in terms of their biocompatibility and influence on enzyme parameters. The obtained l-ASNase on the nGO nanobiocatalyst maintains enzymatic activity and increases its stability, selectively acting on K562 leukemia cells without cytotoxic influence on normal endothelial cells. In the case of treated K562 cells, we confirmed enlargement in the cell and nucleus size, disturbance in the cell cycle (interphase and metaphase), and increased apoptosis rate. The potential therapeutic possibilities of immobilized l-ASNase on leukemia cell damage are also discussed, highlighting the importance of further research in this area for advancing cancer therapy.

## 1 Introduction

l-Asparaginase is an amidohydrolase enzyme (EC 3.5.1.1) that acts through the hydrolysis of l-asparagine to l-aspartate and ammonia. As a result of this activity, the enzyme causes depletion of asparagine, mainly applied in the food and pharmaceutical industries. In food manufacturing, the enzyme purified from different strains of *Aspergillus* has been shown to reduce the buildup of acrylamide in foods such as coffee, biscuits, and potato chips.^1^ Another application of l-asparaginase is its use as an important chemotherapeutic, mainly in hematologic malignancies.^2^

Biological fluids or extracellular environments are sources of asparagine that is essential for cellular protein synthesis and thus for cell proliferation and survival. Moreover, normal cells express the activity of l-asparagine synthetase, which can be upregulated in response to asparagine depletion. In contrast to normal cells, malignant cells lack asparagine synthase activity that disables de novo synthesis of l-asparagine by different cancer cells and makes them susceptible to asparagine deficiency.^2,3^ Recently, there has been renewed interest in metabolic therapies for cancer including amino acid deprivation. l-Asparaginase (l-ASNase) is the basis of contemporary protocols used in the treatment of pediatric acute lymphoblastic leukemia (ALL) and non-Hodgkin lymphomas. Whereas l-ASNase has been used very successfully for over 40 years, the therapy is not free of drawbacks. Recorded side effects include pancreatitis, liver dysfunction, allergic reactions, and general ASNase resistance.^2^ Significant and persistent side effects are more severe in adults, which has contributed to the drug’s infrequent use in adult patients and to the search for novel l-ASNase formulations. Some evidence suggests that additional glutaminase activity of ASNase is correlated with toxic side effects.^4^ On the other hand, it has also been reported that l-ASNase acts effectively in vitro not only against ALL but also against specific subtypes of acute myeloblastic leukemia (AML) or solid tumors, e.g., breast and pancreatic cancer.^5,6^ Since AML is a malignant disease that accounts for ∼70% of acute leukemia cases, further research is still needed to clarify the utility of l-ASNase as an efficient therapeutic compound and to expand its clinical applicability.^2,3,6^

Treatments based on the catalytic activity of enzymes have risen as promising therapeutic tools for different pathologies, from metabolic deficiencies to cancer or cardiovascular diseases.^7^ The key aspect during enzyme-mediated treatment, including l-asparaginase, is to achieve sufficient serum activity and stability of the drug during a particular time period. There are several specified factors influencing l-ASNase activity, including (i) preparation of the enzyme, (ii) l-asparaginase inactivation due to immunological response, and (iii) enzymatic degradation of l-asparaginase by proteases.^3,8^ Main approaches that have already been developed to overcome these challenges for ASNase treatment include PEGylation of an enzyme, conjugation of an enzyme to the cell-penetrating peptide, and enzyme immobilization on nanomaterials/nanoparticles as carriers.^4,9^

The growing research interest in the development of nanostructured materials for therapeutic enzyme immobilization has paved the way for harnessing nanobiocatalytic tools also in modern medicine. In general, enzyme immobilization offers numerous advantages over its free counterparts, such as improved activity, targeting of specific tissues and cells, low immune response, and high retention in the bloodstream.^9,10^ Therefore, immobilized enzymes represent better kinetic properties, more thermal stability, regulated pH tolerance, and excellent storage stability. There are two main types of enzyme immobilization approaches: physical or reversible (physical adsorption, ionic bonding, affinity binding, and metal bonding) and chemical or irreversible (covalent bonding, entrapment, and cross-linking).^9^ The selection of the immobilization method and the supporting material can definitely affect the whole process, e.g., covalent immobilization will prevent leakage of the enzyme from the support, allow easy recovery of the biocatalyst, and facilitate its reuse in consecutive reactions.^11^ Preferably, the support material must prevent enzyme aggregation and denaturation but maintain the native structure of the enzyme, and it should not interfere with the active site.^10^ Graphene oxide (GO) represents the widely used carbonaceous materials that meet these requirements owing to the unique and tunable physicochemical properties, including size, loading capacity, and surface chemistry.^12^ Additionally, the biocompatibility of GO is of undisputable importance for biomedical applications.^13,14^

Despite the growing interest and huge progress in the field of nanobiocatalysts, immobilized asparaginase is not a common solution. There are only scarce reports on asparaginase immobilization, e.g., on gold nanoparticles (GNPs),^5^ carbonaceous materials (GO and nanotubes),^14−17^ chitosan,^18^ or magnetic nanoparticles.^19,20^ The immobilized ASNase on the magnetized nanocomposite demonstrated 2–8 times higher thermostability compared to the free enzyme and showed an extremely extended pH stability range, and therefore it could be a viable option for industrial applications.^20^ Despite some undeniable advantages, the ASNase immobilized on carbon nanotubes has millimolar *K*_m_ values, and optimal activity at pH around 8.0, and no in vitro tests were performed to prove the biocompatibility of such preparations.^16,17^ On the other hand, ASNase conjugated with GNPs has decreased the viability of human breast cancer MCF-7 cells by approximately 60%, at least for the 24 h-incubation, because longer treatment times were not evaluated.^5^ The comprehensive comparison of various preparations of l-ASNase immobilized on a plethora of different supports is presented in Table S1 in the Supporting Information.

Based on these considerations, we describe here the novel and the most effective approach to overcome the limitations of the therapeutic use of l-ASNase–immobilization of l-asparaginase on GO-based supports with high biocompatibility, capacity to maintain the enzyme activity toward l-asparagine, and selectively acting on K562 leukemia cells without cytotoxic influence on normal endothelial cells. We also propose a possible mechanism of immobilized ASNase influence on leukemia cell damage.

## 2 Experimental Procedures

### 2.1 l-Asparaginase Immobilization and Characterization

#### 2.1.1 l-Asparaginase Immobilization

The modified Hummers method was used to prepare GO and nanographene oxide (nGO). Both methods are described in detail in our previously published article.^21^

Lyophilized (with no additives) and purified type II ASNase from *E. coli* was purchased from ProSpec-Tany TechnoGene Ltd. (Israel) and reconstituted according to the manufacturer’s protocol. Immobilization of l-asparaginase on GO and nGO support was performed as follows: the same amount of graphene-derived materials (93 μL solution containing 0.2 mg of nanomaterial) was added to Eppendorf tubes. Then, 0.2 mg of l-ASNase was added to the solutions, and the suspension was made up to a volume of 1 mL with 0.1 M Tris–HCl buffer pH 8.6. The solutions were incubated at 4 °C for 24 h. After equilibrium was achieved, the ASNase-GO/ASNase-nGO complex was pelleted by centrifugation at 10,000 rpm for 10 min at 4 °C. Free l-ASNase concentration in supernatants was measured by the Bradford method to determine the adsorption efficiency. Moreover, the protein desorption rate was determined as follows: after centrifugation of the obtained enzyme-nanomaterial samples, fresh portion of 0.1 M Tris–HCl buffer solution was added to the precipitates. The l-ASNase concentration in the supernatants was measured by the Bradford method after 24, 48, 120, and 168 h (7 days).

For in vitro experiments, the samples of free and immobilized l-ASNase at appropriate concentrations were preincubated with culture medium overnight at 37 °C with gentle shaking.

#### 2.1.2 Microscopic Analyses – AFM

Atomic force microscopy (AFM) images were taken with a Bioscope 2 (Bruker, former Veeco) instrument in the tapping mode. PPP-NCST probes (Nanosensor) were used with a nominal spring constant of 7.4 N/m and a resonance frequency of 160 kHz. The preparations of GO, nGO, and l-ASNase immobilized on both supports were used. All images were collected at a scan rate of 1.0 Hz with a scan resolution of 1024 × 1024 pixels. The raw images were processed with open Gwyddion software.^22^

#### 2.1.3 l-Asparaginase Activity and Kinetic Parameters

The free and immobilized l-ASNase activity was determined using the modified protocol,^23^ by measuring obtained ammonia during reaction with l-asparagine as a substrate. According to this method, 190 μL of appropriate buffer solution [0.1 M simulated body fluid (SBF) pH 7.4 or 0.1 M Tris–HCl pH 8.6] and 10 μL of substrate solution (concentrations in the range from 40 to 189 μmol/mL) were added to tubes. Then, 10 μL of free or immobilized l-ASNase (2.5 IU/mL) was added to start the enzymatic reaction. The mixtures were incubated for 30 min at 37 °C. The process was stopped by adding 10 μL of 1.5 M trichloroacetic acid solution. The released ammonia in the supernatant was determined using Nesler’s method. The concentration of the product was measured at 436 nm using a UV–vis spectrometer. All experiments were performed in triplicate. The same methodology was used to evaluate l-ASNase stability during storage at 4 °C for a maximum of 168 h (7 days).

### 2.2 Effect of l-Asparaginase on Cytophysiology of Human Cell Lines

#### 2.2.1 Cell Culture

HUVEC (human umbilical vein endothelial cells) and K562 (human chronic melogenous leukemia cells) cell lines were used as in vitro models for investigating the cytotoxicity and mechanism of action of studied nanomaterials and enzyme-material conjugates. Adherent HUVECs were cultured on fibronectin-coated dishes in a dedicated endothelial cell medium (ScienCell Research Laboratories, San Diego, USA) containing 10% FBS, 1% endothelial cell growth supplement, and 1% Pen/Strep antibiotics. Suspension growing K562 cells were cultured in TC bottles in RPMI medium with 10% FBS and 1% Pen/Strep antibiotics (Merck, Germany). Both cell lines were grown at 37 °C in a humidified atmosphere with 5% CO_2_.

#### 2.2.2 Viability Assessment – MTT Test

HUVEC cells were seeded to a fibronectin-coated 96-well plate in the amount of 10,000 cells for 24 h before the experiment started. GO, nGO, and their conjugates with l-asparaginase were prepared in concentrations ranging from 0.5 to 500 μg/mL for pure nanomaterials and from 0.1 to 10 IU/mL for the enzyme-nanomaterial nanobiocatalyst. Next, the medium from HUVEC cells was discarded, cells were washed with phosphate-buffered saline (PBS), and samples were added. Cells were incubated for 72 h after which the MTT test was performed. Briefly, sample media were discarded, HUVEC cells were washed twice with PBS buffer to eliminate nanomaterial residuals, and MTT solution (0.5 mg/mL) was added. Cells were incubated for 60 min at 37 °C, after which MTT solution was discarded, cells were washed with PBS buffer, and formazan crystals were dissolved in DMSO. The absorbance of the samples was measured at 570 nm.

In the case of K562 cells, nanomaterial and enzyme-nanomaterial nanobiocatalyst samples at the same concentrations as for HUVEC cells were prepared. The next day, sample media were placed in each well of a U-bottom 96-well plate, and K562 cells in the amount of 10,000 per well were seeded. Cells were incubated for 24 and 72 h after which MTT test was performed. MTT solution was added to each well to the final concentration of 0.5 mg/mL. Cells were incubated for 60 min at 37 °C. Then the plates were centrifuged at 300*g*, MTT solution was discarded, cells were washed with PBS buffer, and formazan crystals were dissolved in DMSO. Plates were centrifuged again, and formazan solution was replaced to F-bottom plates. The absorbance was measured at 570 nm.

#### 2.2.3 Viability Assessment–NRU Assay

GO, nGO, and their conjugates with l-asparaginase were prepared in concentrations ranging from 0.5 to 500 μg/mL for pure nanomaterials and from 0.1 to 10 IU/mL for nanomaterial-enzyme conjugates. The samples were preincubated overnight at 37 °C with gentle shaking. The next day, sample media were placed in each well of a U-bottom 96-well plate, and K562 cells in the amount of 10 000 per well were added. Cells were incubated for 24 and 72 h after which neutral red uptake (NRU) assay was performed. NRU solution was added to a final concentration of 0.033%. Cells were incubated for 120 min at 37 °C, after which cells were counted in a Thoma chamber.

#### 2.2.4 EC50 Calculation

Half-maximal effective concentration (EC50) value was calculated according to the method based upon the principles of a right-angled triangle established by Alexander et al.^24^
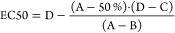


D–concentration of the compound with response over 50%; C–concentration of the compound with response below 50%; A–viability at D concentration; B–viability at C concentration.

### 2.3 Determination of l-ASNase Anticancer Influence

#### 2.3.1 Flow Cytometry

K562 media were supplied with asparaginase or nGO-asparaginase conjugate at a concentration of 5 U/mL and preincubated overnight at 37 °C with gentle shaking. The next day, K562 cells were added and incubated for 72 h. After that, cells were centrifuged for 5 min at 300*g*, resuspended in 1 mL PBS buffer, gently added onto the lymphocyte separation medium (LSM 1077, PAA Laboratories), and centrifuged for 30 min in 300*g* to separate cells and nanomaterial residuals. Subsequently, cell fraction was washed with PBS buffer, resuspended in 70% EtOH, and incubated at −20 °C overnight. Then, K562 cells were washed twice in PBS buffer and incubated with PI (20 μg/mL) and RNase A (50 μg/mL) for 60 min at 37 °C in the dark. Fluorescence intensity was measured using a BriCyte E6 flow cytometer (Mindray, Shenzhen, China), with 488 nm excitation and 585 nm emission wavelengths. Data were analyzed using FCS Express 7 (De Novo Software, USA).

#### 2.3.2 Fluorescent Microscopy

For analyses, K562 cells were fixed with 4% (v/v) in 0.1 M PBS (pH 7.2) for 1 h at room temperature. Then, the fixed samples were washed and transferred to microscope slides. Finally, DNA was stained with 1 mg/mL Hoechst 33342 (Molecular Probes) and covered with ProLong Gold Antifade Mountant (Life Technologies). The slides were observed using light and fluorescence microscopy in a Nicon Eclipse 80i. The percentage of cells was calculated on the basis of three independent experiments, during which 100 cells were counted for each variant and divided into subgroups corresponding to different cell status. Results were photographed using a Nikon DS-5Mc color cooled digital camera and NIS - Elements AR 3.00 image analysis software; representative images are presented in the manuscript.

## 3 Results

### 3.1 l-Asparaginase Immobilization and Characterization

**Figure 1:**
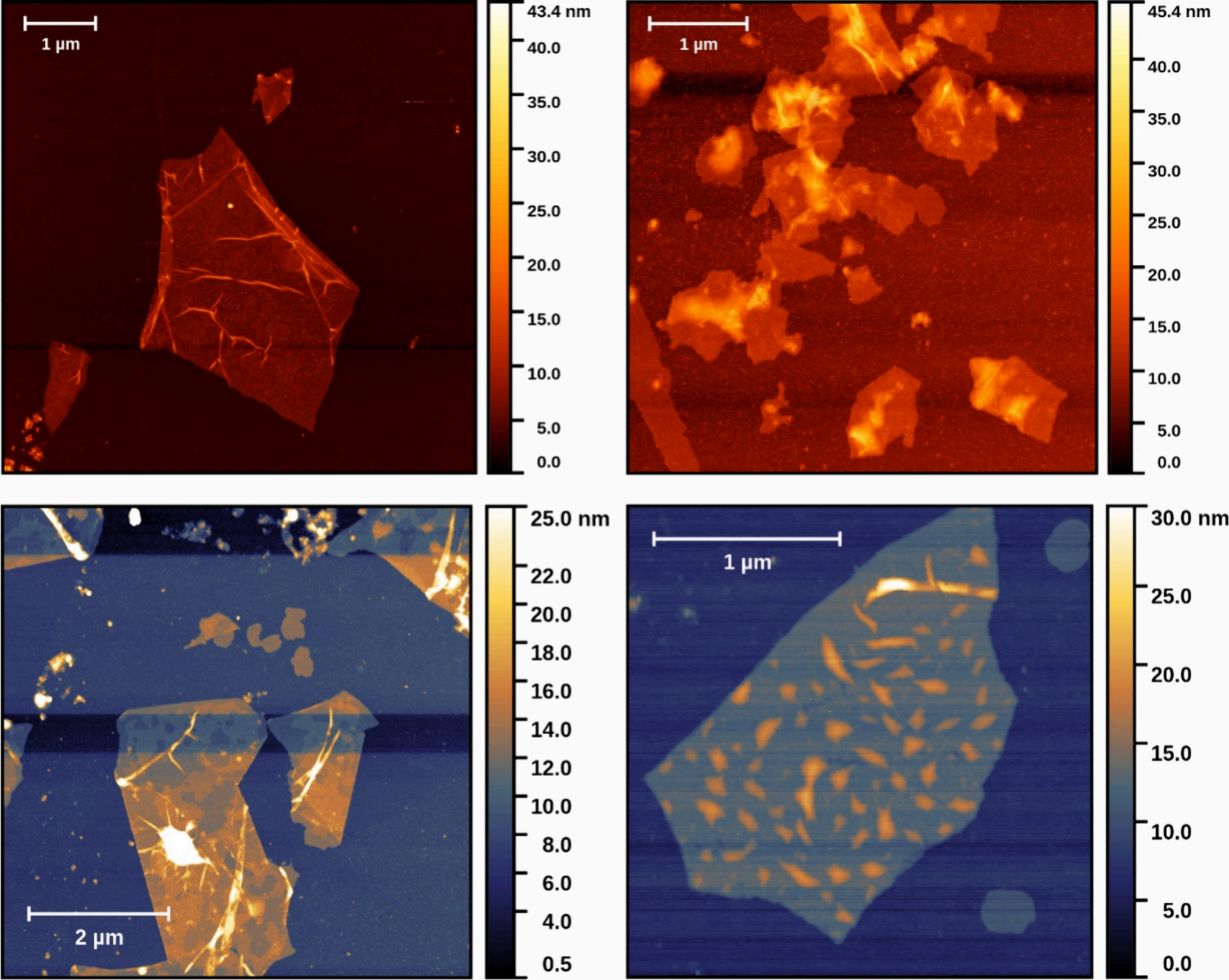
AFM imaging of GO (left) and nGO (right) before (upper) and after (bottom) l-ASNase adsorption. Separate “islets” of the immobilized enzyme are visible on the nGO surface (bottom right panel), which confirms the effective immobilization.

In the first set of experiments, we aimed at confirming the suitability of GO and nGO for the enzyme immobilization process. According to the previously published results,^21^ large GO sheets (sized approximately several micrometers) of atomic thickness were cut to the size of hundreds of nanometers as a result of ultrasonic processing. Naturally occurring wrinkles on the GO surface are the places where the process is most effective (Figure 1).

l-ASNase was then immobilized through adsorption on the surfaces of both GO and nGO under the same conditions. The adsorption process was very efficient and stable; 100% of enzymatic protein adsorbed at the surface of GO and nGO materials, and no desorption was observed within 7 days, as determined through the protein concentration assay. We also confirmed the adsorption of l-ASNase with AFM imaging (Figure 1, bottom panel). Enzyme immobilization on both surfaces (GO and nGO), although quantitatively similar, is qualitatively slightly different. While l-ASNase forms a rather compact layer on the GO surface, clear “islets” of the immobilized enzyme are visible on the nGO surface. Using the AFM phase mode, we were able to distinguish the enzyme particles and the supporting matrix as two separate phases (Figure S1).

**Figure 2:**
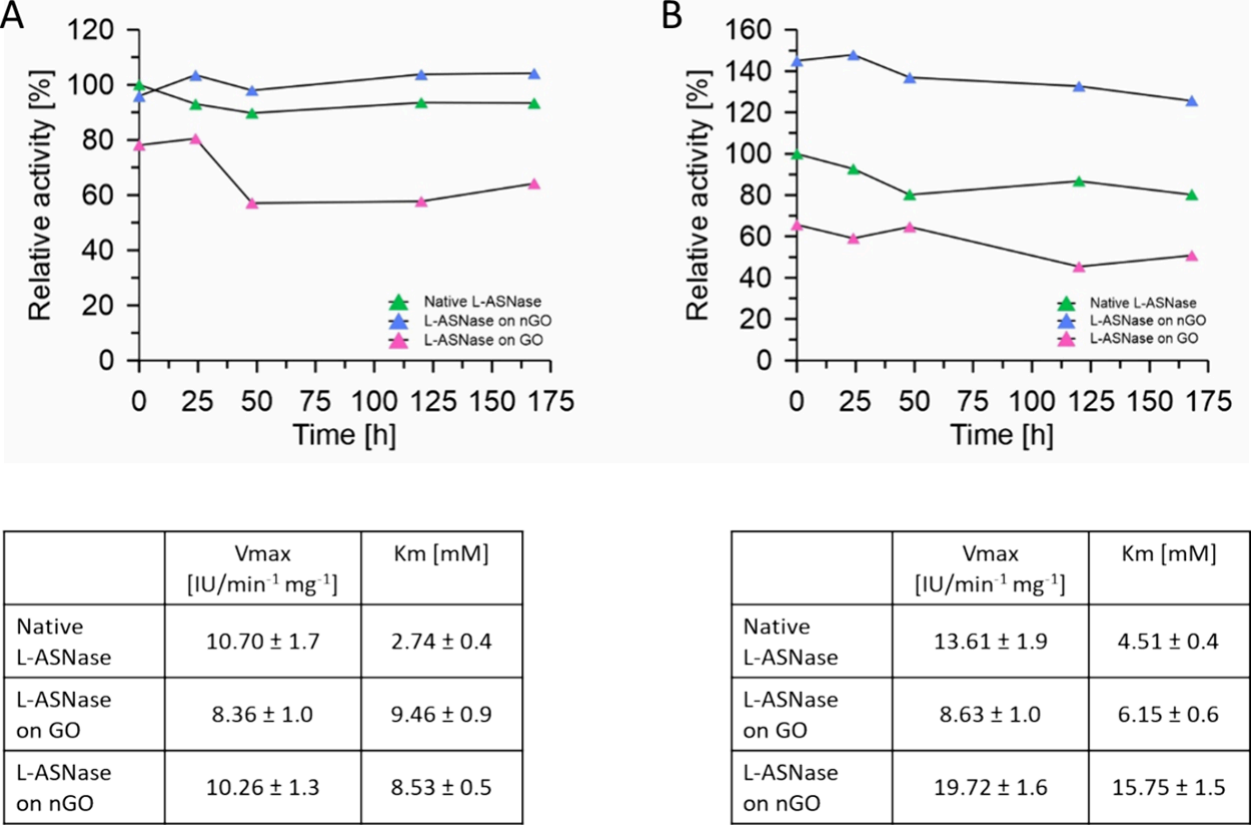
Determination of kinetic parameters and stability of the l-ASNase activity during storage: (A) Assays performed in SBF pH 7.4, which reflects the composition of human plasma. B) Assays performed in Tris–buffer pH 8.6 which is optimal for l-ASNase activity. For stability experiments, the initial activity of native ASNase was taken as 100% control. For kinetic parameters, the values are presented as mean ± SD (for *n* = 3).

It is commonly known that immobilization stabilizes the tertiary and quaternary structure of the enzymatic protein.^10^ In these terms, the evenly dispersed islet-like structures seem to be more beneficial compared to the flat and disorderly deposition of l-ASNase on the GO surface. This phenomenon underlies also the different activity and stability (Figure 2) of both obtained biocatalytic systems.

The enzyme immobilization was performed in Tris–HCl buffer pH 8.6, and activity determination toward asparagine was comparably done in pH 7.4 (simulated body fluid, SBF, as a solution better reflecting the body fluids environment) and pH 8.6 (Tris–HCl buffer at pH optimum for l-ASNase, Figure 2). Accordingly, the enzyme activity is well maintained when immobilized on nGO and kept in SBF, whereas in Tris–HCl, the enzyme-nGO conjugate is highly active with more labile activity but still higher than in the case of free enzyme.

### 3.2 Effect of l-Asparaginase on Cytophysiology of Human Cell Lines

Both the GO-based materials seem to be beneficial for ASNase immobilization and for maintaining its enzymatic activity. In the next set of experiments, we checked the viability of normal human endothelial cells, HUVECs, cultured in the presence of GO and nGO. We performed two widely accepted viability tests: MTT assay reflecting the metabolic activity (Figure S2 in the SI) and NRU test for lysosomal functionality (Figure S3 in the SI).

Based on the MTT assay results, we calculated EC50 value for 376.19 μg/mL for GO and 26.64 μg/mL for nGO. Thus, we can conclude that nGO is tolerated by HUVECs only at low (up to 26 μg/mL) concentrations for 72 h, whereas the number of 50% viable cells based on lysosomal functions (concluded from the NRU assay) is maintained up to 434 μg/mL of nGO for 72 h.

Both materials were also tested in terms of their influence on the viability of leukemia cells–K562. Again, we performed two different viability tests: MTT and NRU (Figures S4 and S5, respectively, in the SI). Different concentrations of GO and nGO up to 250 μg/mL do not aggravate the leukemia cells viability similarly in both tests, and the EC50 values for GO and nGO were over the concentration of 500 μg/mL. The comparison of cytotoxic effect of GO and nGO toward both tested cell lines, based on the results of MTT and NRU tests, is shown in Table 1.

**Table 1:** Comparison of Cytotoxic Effect of the Tested Materials and Nanobiocatalysts Expressed as Half-Maximal Effective Concentration (EC50) after 72 h of Incubation with Two Different Cell Viability Tests (MTT and NRU Assays)^a^

viability test	material tested	EC50 value	
		HUVEC cells	K562 cells
MTT assay	GO [μg/mL]	376.19 ± 29.03	>500
	nGO [μg/mL]	26.64 ± 2.74	>500
	Free ASNase [IU/mL]	3.41 ± 0.31	8.96 ± 0.97
	ASNase on GO [IU/mL]	4.01 ± 0.59	>10
	ASNase on nGO [IU/mL]	4.84 ± 0.42	>10
NRU assay	GO [μg/mL]	473.84 ± 48.60	>500
	nGO [μg/mL]	434.51 ± 40.81	>500
	Free ASNase [IU/mL]	5.93 ± 0.47	2.20 ± 0.21
	ASNase on GO [IU/mL]	8.87 ± 0.79	5.20 ± 0.51
	ASNase on nGO [IU/mL]	>10	1.85 ± 0.19

a The values are presented as mean ± SD (for *n* = 4).

**Figure 3:**
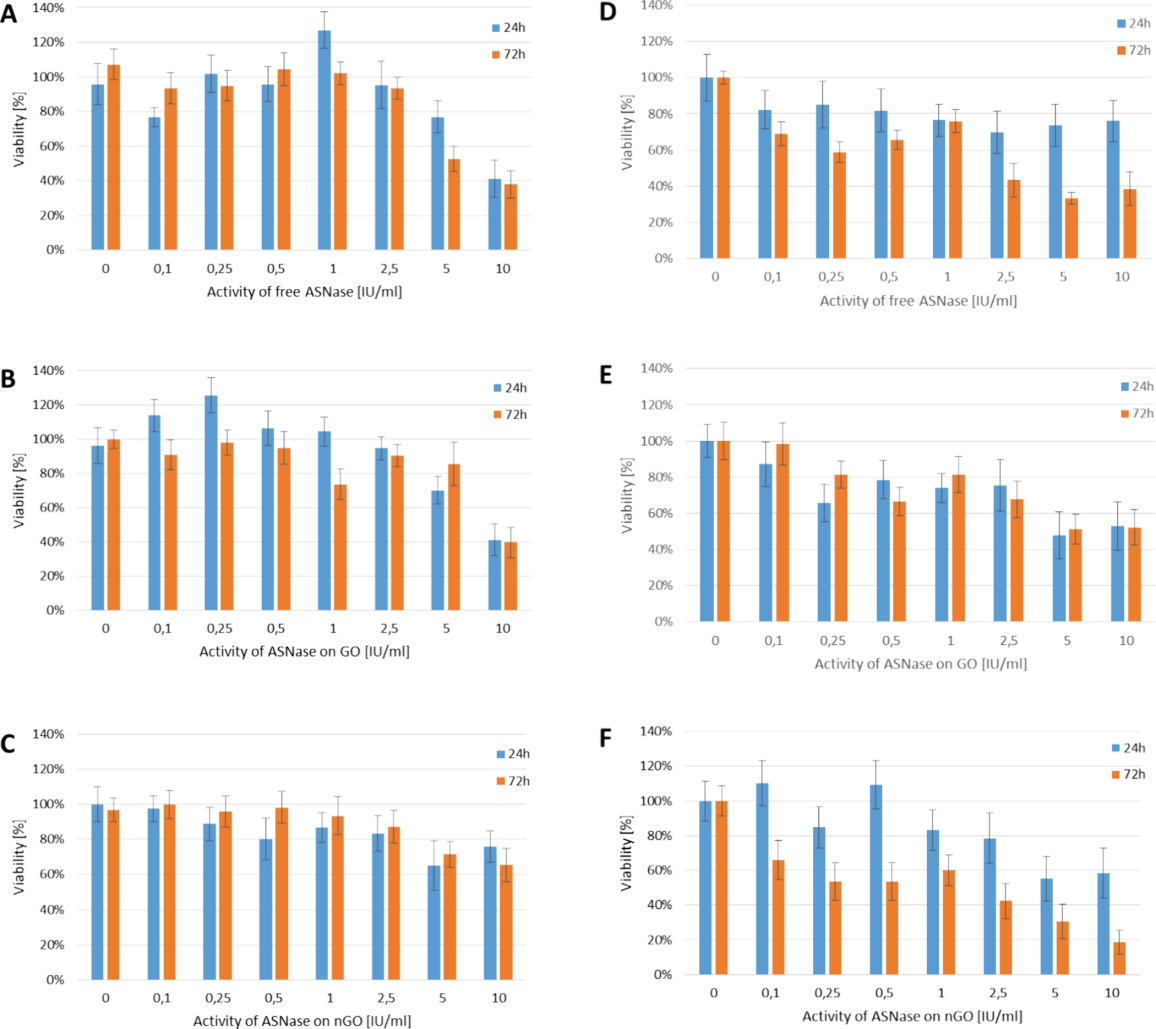
Comparison of HUVEC and K562 cell viabilities treated with free and immobilized ASNase: (A–C) NRU assay on HUVEC cells. (D–F) NRU assay on K562 cells in relation to different l-ASNase activities from 0.1 to 10 IU/mL. In the case of immobilized enzyme, these activities correspond with 0.5, 1.25, 2.5, 5.0, 12.5, 25, and 50 μg/mL of support. The values are presented as mean ± SD (for *n* = 4).

Furthermore, the functional activity of l-ASNase in three forms, free, immobilized on GO, and on nGO, was tested in HUVEC and K562 cell cultures (Table 1). MTT assay reflecting the cell metabolism showed that l-ASNase, immobilized or not, decreases the normal cells viability below 70% of control only at the highest tested activities 5 and 10 IU/mL (Figure S6A–C), and at the same time, it has no influence on leukemia cell viability (Figure S6D–F). As the MTT test reflects the metabolic activity and glycolytic NADH production efficiency, it poorly correlates with the viable cell number in the case of leukemia cells due to metabolic reprogramming. Hence, we assayed the cell viability with the NRU test that reflects the lysosomal activity, and it clearly indicated the differences between HUVECs and K562 cells. Collective results for 24 and 72 h of incubation at different ASNase forms and activities are presented below (Figure 3).

Based on the above results, it is clear that the most promising anticancer activity of l-ASNase is observed for the enzyme immobilized on nGO as we can achieve the EC50 after 72 h at a concentration as low as 1.85 IU/mL, which is completely nontoxic for HUVEC cells (with EC50 > 10 IU/mL). In comparison with free ASNase, this nanobiocatalyst is slightly more efficient after 72 h of incubation, whereas in comparison with ASNase on GO, it is not only more active but also stable (see Figure 2A for stability determination).

### 3.3 Determination of l-ASNase Anticancer Influence

**Figure 4:**
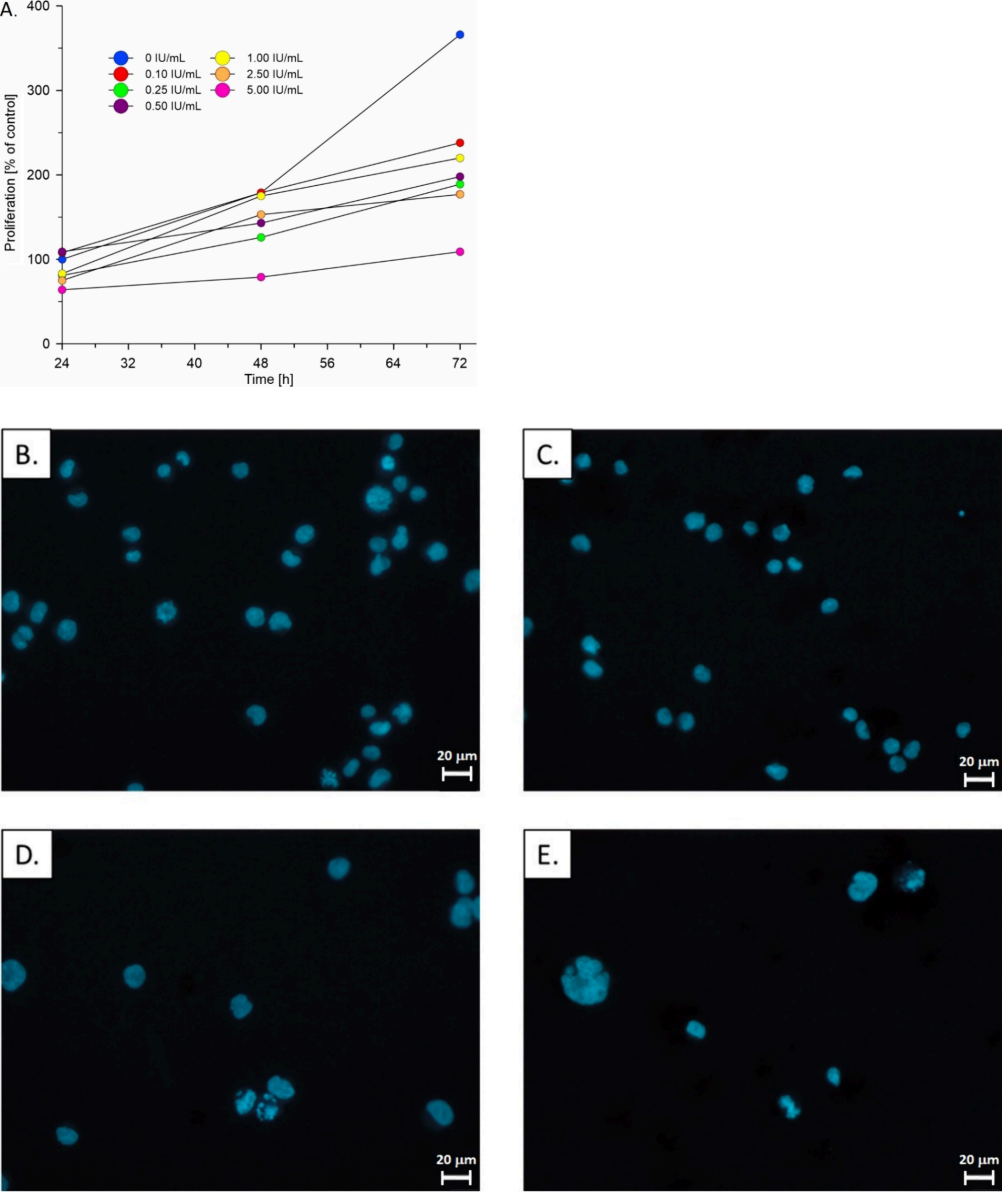
A. Proliferation rate of K562 cells treated with different concentrations of l-ASNase on nGO. The total number of viable cells was calculated after Trypan blue staining. The number of cells after 24 h in the untreated control was taken as 100%. (B–E) Representative fluorescent microphotographs of cells stained with Hoechst showing the decrease in cell number and their morphological changes (B, control cells; C, cells treated with nGO; D, cells treated with l-ASNase; E, cells treated with 5 IU/mL ASNase on nGO; all treatments for 72 h). Scale bar is 20 μm.

To undisputably confirm the benefits of immobilized ASNase on nGO, we performed an analysis of K562 proliferation rate, which showed the enzymatic activity-dependent decrease in proliferation in the presence of l-ASNase immobilized on nGO (Figure 4A). Activity of 5 IU/mL ASNase on nGO strongly influenced the cell morphology, and it can be concluded that enlarged cells are one of the hallmarks of these alterations (Figure 4E).

**Figure 5:**
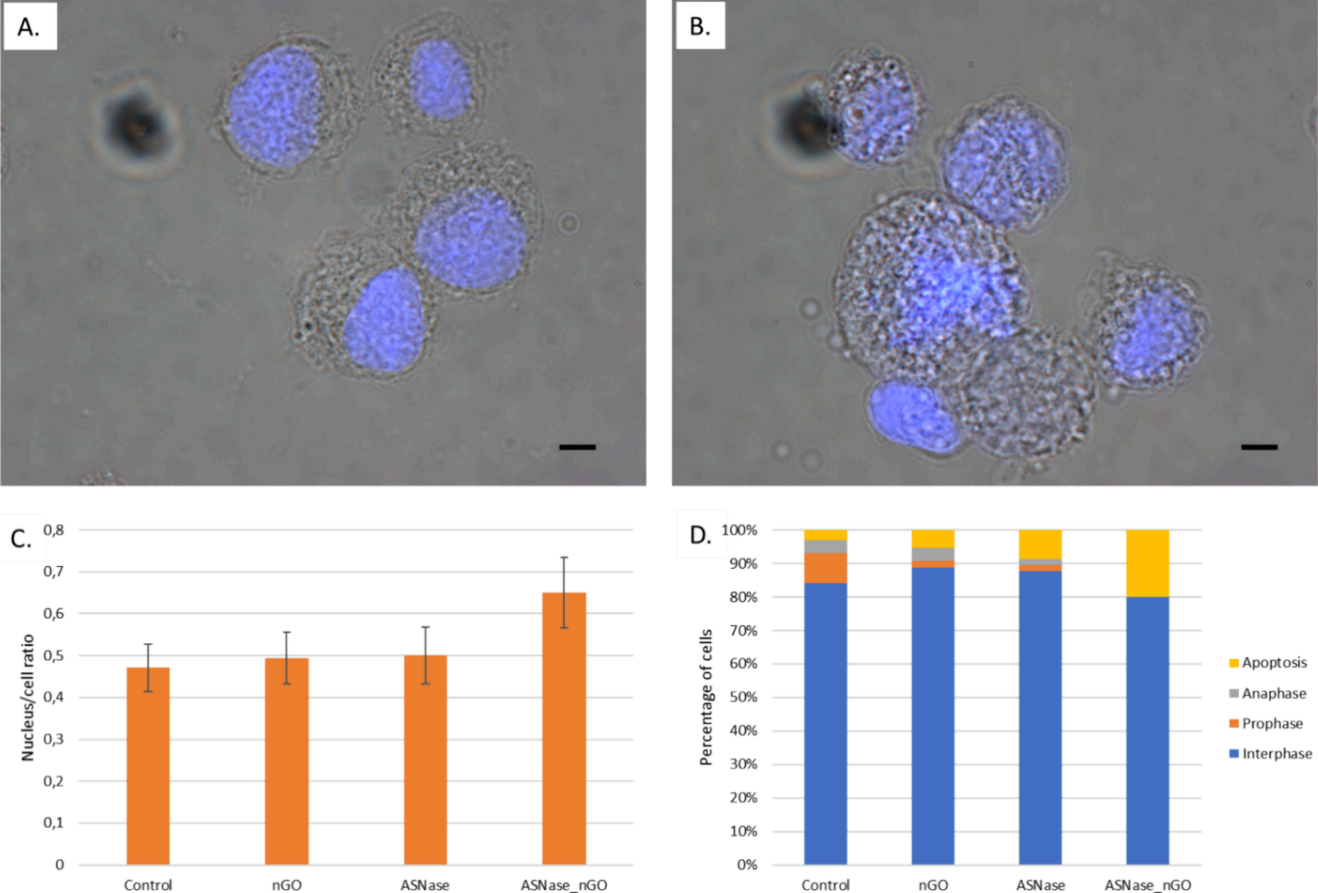
Changes in the K562 cell morphology and cell cycle. Merged representative images of bright-field and fluorescent microscopy (cells stained with Hoechst) show untreated K562 cells (A) and cells treated with 5 IU/mL l-ASNase on nGO (B). Scale bar represents 50 μm. The measured nucleus-to-cell ratio (C) and percentage of apoptotic cells (D) increase in cells treated with 5 IU/mL l-ASNase on nGO after 72 h-treatment was calculated for 100 cells for each variant. The values are presented as mean ± SD (for *n* = 3).

Additional experiments using flow cytometry analysis (Figure S7) and electron microscopy combined with cell and nucleus size determination (Figure 5) indicated the potential mechanisms involved in K562 cell damage, such as the enlargement of the cell and nucleus size, disturbance in cell cycle (interphase, metaphase), and inducing apoptosis.

Changes in the cell cycle indicate a minor decrease in the cells in the G1 phase and an increase in the G2 phase of the cell cycle (Figure S7). However, these alterations are slightly expressed, and they do not justify the observed disturbance of cell proliferation. We obtained a more detailed insight into these mechanisms after analysis of electron microscopy data (Figure 5). The enlarged cells with a not well-defined nucleus, chromatin dispersion, and cell division disorders are the main morphological features appearing in the treated cells. In the case of treatment with l-ASNase on nGO, the percentage of apoptotic cells rises over 20%.

## 4 Discussion

Systemic administration of bacterial l-ASNase is used to eliminate rapidly proliferating cancer cells with a high demand for exogenous asparagine through lowering the bioavailability of this essential amino acid. For therapeutic applications, type II l-asparaginases from *Escherichia coli* or *Erwinia chrysanthemi* which have a strong preference for l-asparagine as a substrate exhibit a low glutaminase side activity (0.1–2%) and are comparatively easy to produce and are mainly used.^3^ Therefore, we harnessed an *E. coli*l-ASNase type II preparation to develop a strategy of enzyme immobilization on a GO support. GO-based nanobiocatalyst with adenylate kinase activity was previously studied by our group^25,26^ for its ability to maintain the nucleotide balance in the extracellular environment of mesenchymal stem cells and lung adenocarcinoma A549 cells. Deciphering the AK-GO role in the extracellular environment was underpinned with the alterations in enzyme kinetic parameters: increased and stable activity as well as decreased *K*_M_ value toward both substrates, ADP and ATP.^26^ Here, we compared GO with nGO in terms of their suitability to serve as support for another important enzyme, l-ASNase, for immobilization. l-ASNase treatment is often limited by its short circulatory half-life and undesired side effects; therefore immobilization is suggested as one of the ways to overcome the limitations.^9,10,14^

Catalytic parameters of an enzyme are very important to conclude on the enzyme behavior after immobilization. We have determined the maximum velocity (*V*_max_) and Michaelis constant (*K*_M_) in Tris–buffer pH 8.6 and in SBF that better reflects the physiological environment in human tissues; however, its pH 7.4 is a little beyond the optimal pH for asparaginases. The maximum velocity of l-ASNase immobilized on GO decreases compared with the free enzyme, while immobilization on nGO maintains a *V*_max_ value at pH 7.4 and increases it at pH 8.6. The *K*_M_ value of wild-type l-ASNase II from *E. coli*, as determined by different groups, was ranging from 0.4 to 9.2 mM,^14,15,19,20,27^ and it was mainly increasing after immobilization. *K*_m_ shows the affinity of an enzyme to its substrate, and its increase with immobilization means lower affinity to the substrate due to steric hindrances. Our results stay in agreement with these observations. Additionally, the high enzymatic activity after immobilization on nGO was maintained in SBF at the same level for 7 days. It stays in good agreement with the majority of results obtained by different research groups for l-ASNase immobilized on various supports (for comparison see Table S1 in the SI).

Therapeutic application of immobilized enzyme requires meeting specific needs: specificity, stability, biocompatibility, and hemocompatibility.^28^ Supports developed so far for ASNase immobilization were only partially characterized, and these studies had some serious shortcomings as indicated in the introduction. In our set of results, we present comprehensive biocompatibility data: the GO-based supports as well as immobilized L-ASN-ase were added in a wide range of concentrations to normal human endothelial cells (HUVECs) and to K562 leukemia cells. MTT assay underpinned with the metabolic activity of the cells was the first choice assay; however, it gave us surprising results. The HUVEC metabolic activity was lower in comparison to the NRU assay, and K562 cells exhibited a high capacity to reduce MTT in control cells as well as in the presence of compounds added to the cell medium. Definitely, dysregulated metabolism is one of the hallmarks of cancer and can lie in the background of observed outcomes, and another reason is the high reductive capability of cells.^29^ Thus, we harnessed the NRU assay as the second most used test for cell viability. There are negligible differences in cytotoxic influence of free and immobilized l-ASNase on nGO toward K562 cells; however, the viability of HUVEC cells in the presence of l-ASNase on nGO is significantly higher. The enhanced viability and resistance of normal healthy cells to the applied anticancer treatment are promising in terms of potential side effects and disturbances in healthy cell functions.

As outlined in the introduction, using bacterial type II enzymes in clinical protocols also has another disadvantage, the immunological response. Problems with immunological activity can be mitigated, however, by attaching poly(ethylene glycol) (PEG) polymers onto surface lysines of the protein. The resulting PEGylated proteins are highly hydrated, with two or three waters solvating each ethylene glycol unit, increasing both the size and the hydrophilicity. Of course, this chemical modification strategy does have drawbacks. One of them is that the attachment of the linker–PEG conjugate proceeds in a random fashion. Moreover, PEGylated protein can also refuse a substrate from the active site of the protein.^30^ In our approach, enzyme immobilization by physical adsorption on the GO surface allows one to maintain the shape and function of the active site and to overcome disadvantages connected with immunogenicity by hiding some potential epitopes of the enzyme. The immobilized protein, together with additional molecules that can potentially be adsorbed on the support, will prevent the interactions with antibodies or blood cells.^31^ With regard to the hemocompatibility of the nanobiocatalyst in blood circulation, we have described some aspects in recently published papers from our group [21 (for nGO), 26 (for GO)]. Feng and colleagues reported hemolytic effects induced by GO above 10 μg/mL; however, also lower hemolytic concentrations have been reported by other groups.^32,33^ In vitro studies on pristine graphene and GO provided evidence that concentrations up to 75 μg/mL do not interfere with platelet function or the pathways of plasma coagulation and that GO, up to 50 μg/mL, does not interfere with platelet aggregation or fibrinogen polymerization.^33^ Studies on complement activation and interactions of GO and other cells circulating in the blood have reported inconsistencies in GO effects as summarized in the review by Palmieri et al.^31^ Bearing in mind the benefits of using GO and specifically nGO as an immobilization support, we assume that novel nanobiocatalysts will be developed in the nearest future. Although asparaginase has been an important chemotherapeutic agent for decades, until recently, its use was limited to pediatric hematology. We believe that our research results will draw attention to the enormous potential of immobilized l-ASNase and pave the way for further comprehensive research.

## 5 Conclusions

In the presented study, we prove experimentally that nGO is a much better support than the unmodified pristine material, GO, in terms of l-ASNase immobilization for therapeutic purposes. The obtained nanosized GO is biocompatible toward human cells, which, together with maintaining the stable enzymatic activity of l-ASNase, makes it a promising therapeutic strategy. In the case of leukemia K562 cells, we confirmed the enlargement in the cell and nucleus size, disturbance in the cell cycle (interphase, metaphase), and increased apoptosis rate after treatment with immobilized l-ASNase with 5 IU/mL activity. Therefore, the nGO-based nanobiocatalyst with l-ASNase selective activity toward leukemia cells represents a promising example of harnessing the nanostructured materials to develop novel anticancer treatment modalities.

## Supporting Information Available

Additional experimental data, including results of cytotoxicity evaluation and cell cycle analysis through flow cytometry; comparison of various preparations of l-ASNase immobilized on different supports; AFM phase image of l-ASNase on nGO sample; concentration-dependent viability of HUVECs treated with GO and nGO and assayed with MTT test and NRU test; concentration-dependent viability of K562 cells treated with GO and nGO and assayed with MTT test and NRU test; MTT viability assessment of HUVEC cells and K562 cells; and cell cycle analysis through flow cytometry (PDF)
