# Supplementary material for: l-Asparaginase Immobilized on Nanographene Oxide as an Efficient Nanobiocatalytic Tool for Asparagine Depletion in Leukemia Cells
Source: Bioconjug Chem. 2025 Jan 14;36(2):253–62. doi: 10.1021/acs.bioconjchem.4c00518 (PMC11843607; doi:10.1021/acs.bioconjchem.4c00518)
Supplement: Supplementary file 1 — bc4c00518_si_001.pdf [file bc4c00518_si_001.pdf]

SUPPLEMENTARY INFORMATION

L-asparaginase immobilized on nano-graphene oxide as an efficient nanobiocatalytic tool for asparagine depletion in leukemia cells

*Paulina Erwardt[1], Bartosz Szymczak[2], Marek Wiśniewski[1], Bartosz Maciejewski[3], Michał Świdziński[4], Janusz Strzelecki[5], Wiesław Nowak[5], Katarzyna Roszek[2]**

[1] Department of Materials Chemistry, Adsorption and Catalysis, Faculty of Chemistry, Nicolaus Copernicus University in Torun, ul. Gagarina 7, 87-100 Torun, Poland

[2] Department of Biochemistry, Faculty of Biological and Veterinary Sciences, Nicolaus Copernicus University in Torun, ul. Lwowska 1, 87-100 Torun, Poland

[3] Department of Immunology, Faculty of Biological and Veterinary Sciences, Nicolaus Copernicus University in Torun, ul. Lwowska 1, 87-100 Torun, Poland

[4] Department of Cellular and Molecular Biology, Faculty of Biological and Veterinary Sciences, Nicolaus Copernicus University in Torun, ul. Lwowska 1, 87-100 Torun, Poland

[5] Department of Biophysics, Institute of Physics, Faculty of Physics, Astronomy and Informatics, Nicolaus Copernicus University in Torun, ul. Grudziądzka 5, 87-100 Torun, Poland

* corresponding author: kroszek@umk.pl

Table S1. Comparison of various preparations of L-ASNase immobilized on different supports (compilation of literature data).

Support material	Amount of immobilized L-asparaginase	Stability			Reusability	Cytotoxicity	Ref.
		Storage	pH	Thermal			
Chloro-Modified Magnetic Fe_3O_4@MCM-41	-	Immobilized L-ASNase maintained 47 and 32.5% of initial activity after 4 weeks of storage at 4°C and 25°C, respectively, whereas the activities of the native L-ASNase remained at 39.2% and 16% level	85% of initial activity in a wide pH range of 7.0–9.0	After incubation at 55°C for 150 min, the immobilized L-ASNase retained 75.4% of initial activity, and native L-ASNase 60.4%. Native L-ASNase was inactivated after 180 min, immobilized L-ASNase still had a higher catalytic activity (69.7% of initial).	Immobilized L-ASNase maintained nearly 81% of its initial activity in the first 5 cycles, more than 62% activity after 10 cycles, and 42.2% activity after 18th reuse	-	[1]
Ca-ALG/MWCNT-COOH	187.5 IU on 10 mg of MWCNT-COOH	Under storage conditions (4 weeks at 30°C), the immobilized and native enzyme retained 81.2 and 71.4% of initial activity	Optimal pH raised to 8.5	Ca-ALG/MWCNT-COOH/L-ASNase showed higher activity than the native L-ASNase at temperature range 45-65°C	After 5, 10 and 14 cycles, the immobilized L-ASNase retained 58.7%, 37.1% and 36.4% of the initial activity	-	[2]
Pristine MWCNTs or functionalized with different HNO_3 concentrations	The maximum adsorption capacity at ASNase concentration of 1.5×10^{-3} g mL^{-1} for all the MWCNTs used	-	-	-	No loss of immobilized ASNase activity during 6 consecutive reaction cycles	-	[3]
Magnetic poly(HEMA-GMA) nanoparticles	66.43 mg/g nanoparticle	Immobilized asparaginase maintained 30% of initial activity after 40 days of storage	pH optimum change from 7.5 for native L-asparaginase to 6.5 for immobilized enzyme	Optimum temperature change from 45°C for native L-asparaginase to 55°C for immobilized L-asparaginase	Immobilized L-asparaginase lost only 15% of initial activity after 8 cycles of use	-	[4]
Fe_3O_4-NH_2-4VPBA-PEG	200 IU of enzyme per 0.1g of Fe_3O_4-NH_2-4VPBA-PEG NPs. The immobilization yield = 94 %	Native and immobilized ASNase stored at 25°C for 7 days exhibited 70% and 90% of initial activity, respectively, and after 28 days - both forms have activity around 50%	pH optimum change from 8.5 for native L-asparaginase to 7.5 for immobilized enzyme	Native L-ASNase retained only 9% of initial activity at 60 °C, 120 min, whereas immobilized enzyme retained over 25% of initial activity	Immobilized enzyme retained 54% of initial activity after 10 cycles		[5]
Chitosan nanoparticles	Obtained immobilization efficiency was 90% for all nanoparticles. Chitosan	Immobilized enzyme retained more than 80% of initial activity after storage for 9 months at room temperature	pH stability range 6.5 - 9.5	The temperature stability range 25 - 65°C (30-35°C for native enzyme)	-	IC50 for various cell lines was established: A549 ~25 µg/mL MCF7 ~27 µg/mL HTC116 ~22 µg/mL	[6]

	nanoparticles were the fastest in reaching the maximum immobilization efficiency after 72h					Jurkat E6.1 under 20 μg/mL	
Gold nanoparticles		Immobilized enzyme retained more than 65% of initial activity after storage for 9 months at room temperature	pH stability range 7 - 9	The temperature stability range 25-50°C	-	A549 ~40 μg/mL<br>MCF7 ~60 μg/mL<br>HTC116 under 50 μg/mL<br>Jurkat E6.1 under 30 μg/mL	
Magnetic Iron (III) Oxide nanoparticles		Immobilized enzyme retained more than 65% of initial activity after storage for 9 months at room temperature	pH stability range 7 - 9.5	The temperature stability range 25-45°C	-	A549 ~40 μg/mL<br>MCF7 ~50 μg/mL<br>HTC116 ~40 μg/mL<br>Jurkat E6.1 ~22 μg/mL	
Silver nanoparticles		Immobilized enzyme retained more than 65% of initial activity after storage for 9 months at room temperature	pH stability range 7 - 9	The temperature stability range 25-50°C	-	A549 ~45 μg/mL<br>MCF7 ~60 μg/mL<br>HTC116 ~55 μg/mL<br>Jurkat E6.1 ~35 μg/mL	
Silica nanoparticles		Immobilized enzyme retained more than 65% of initial activity after storage for 9 months at room temperature	pH stability range 7 - 9	The temperature stability range 25-45°C	-	A549 ~40 μg/mL<br>MCF7 ~55 μg/mL<br>HTC116 ~50 μg/mL<br>Jurkat E6.1 ~35 μg/mL	
GO-Asp-Fe_3O_4 nanocomposite	-	-	Native and immobilized enzyme exhibit pH stability range 5 - 9.At pH=3 native enzyme lost its activity, whereas immobilized form retained 75% of initial activity	Stable for up to 1 h at 50 °C. After 1h at 60 °C immobilized enzyme retained 21% of initial activity (native enzyme ~6%)	Immobilized enzyme retained 54% of initial activity after 8 cycles	-	[7]
Functionalized Fe_3O_4@MCM-41	50 IU L-ASNase on 5mg of Fe_3O_4@MCM-41 powder	Immobilized enzyme retained 65% of initial activity after 30-days storage at 4 °C, and 53% at 25 °C	At pH 9 immobilized enzyme has 81% of initial activity, whereas native form has 73%	Native enzyme lost 70% of initial activity after 3 h incubation at 50 °C, under the same conditions immobilized enzyme still retained 94% activity	Immobilized enzyme retained 63 % of initial activity after 16 cycles	-	[8]
p(HEMA-GMA) cryogels	100 IU L-ASNase on 100 mg cryogel, immobilization yield 69%	After 4 weeks, the native L-ASNase lost more than 72% of the original activity, whereas p(HEMA-GMA)/L-ASNase lost 56% of initial activity	-	The native enzyme retained 18% of initial activity after 3 h incubation at 60 °C for 3 h. The conjugate retained 66% of its original activity under the same conditions	p(HEMA-GMA)/L-ASNase conjugate maintained 52% of initial activity after 10 cycles	-	[9]
Nanogels of PEG-grafted poly	-	-	Immobilized enzyme pH stability ranged 6.5 - 12 is comparable with native	Immobilized ASNase lost about 10 % of enzyme activity after 1 h incubation	-	-	[10]

HPMA with bis (α-cyclodextrin)			form, the native enzyme activity is strongly reduced below pH 5.0	at 50 °C, after 1h incubation at 60 °C the immobilized enzyme activity decreased to 39 % and 56 % (on P(HPMA-MPEGA) and bisCD nanogels, respectively), and after 1h at 70 °C – to 34 % and 47 % of initial activity			
Carboxymethyl dextran	15 mg of enzyme at the optimum molar ratio of ASNase/CMD equal to 1:85	-	More resistant to pH changes after immobilization	Stable activity during 30 min of incubation at temperatures 30 – 95 °C	-	-	[11]
Functionalized Carbon Xerogels	200 µL ASNase solution (from 0.02 to 0.38 g/L, or 4.5 to 85.5 IU/mL) added to 2 mg of each CX. The optimal conditions (81 min of incubation, pH 6.2, and 0.36 g/L of L-ASNase) yielded 100% efficiency	After 10 days of incubation at 4°C and 25°C, the bioconjugate retained 85% and 91% of initial activity.	After 120 minutes of incubation at pH 5-8 modified enzyme showed almost no activity loss	-	The ASNase/CX bioconjugate maintained 97% initial activity after 6 cycles of use	-	[12]
Nanogels of PEG-grafted poly-HPMA and bis(α-cyclodextrin)	-	-	-	-	-	The IC50 (for HL60 cells) values for native ASNase, poly-(HPMA-MPEGA)-ASNase, and bisCD-ASNase nanogel were calculated as 1.31, 2.17 and above 3 IU/ml, respectively	[13]
Tubular poly(3,4-ethylene dioxythiophene)-polypyrrole/Ni/ Pt (PEDOT-PPy-COOH/Ni/Pt) micromotors	500 µL of asparaginase solution (0.3 mg/mL) was added to 200 µL of micromotors suspension	After 45 days storage at 4 °C, the immobilized L-ASNase maintained almost 45 % of initial activity	-	After 200 minutes of incubation at 55 °C immobilized L-ASNase maintained 60% of initial activity, whereas free L-ASNase exhibited 27 % of initial activity	The immobilized L-ASNase retained 60% of initial activity after 5 cycles of use	-	[14]
Amino-epoxy-agarose	The enzyme was immobilized at a ratio 1:10 (m:v) of support to the enzyme solution (6.7 mg/mL)	After 60 days storage at 4 °C, the immobilized L-ASNase maintained 82% of initial activity vs. 60% for native enzyme	Stable activity after 60 minutes of incubation at pH 4-10	After 60 minutes of incubation at 60 °C immobilized L-ASNase showed 20% of initial activity	The L-ASNase immobilized on amino-epoxy-agarose support maintained ~70% of initial activity after 7 cycles of use	-	[15]

References:

[1] Ulu, A., Noma, S.A.A., Koytepe, S., Ates, B. Chloro-Modified Magnetic Fe_3O_4@MCM-41 Core-Shell Nanoparticles for L-Asparaginase Immobilization with Improved Catalytic Activity, Reusability, and Storage Stability. Appl Biochem Biotechnol. 2019; 187(3), 938-956.

[2] Ulu, A., Karaman, M., Yapıcı, F., Naz, M., Sayın, S., Saygılı, E. İ., Ateş, B. The Carboxylated Multi-walled Carbon Nanotubes/l-Asparaginase Doped Calcium-Alginate Beads: Structural and Biocatalytic Characterization. Catal Lett 2019; 150, 1679 - 1691.

[3] Almeida, M.R., Cristóvão, R.O., Barros, M.A., Nunes, J.C.F., Boaventura, R.A.R., Loureiro, J.M., Faria, J.L., Neves, M.C., Freire, M.G., Santos-Ebinuma, V.C., Tavares, A.P.M., Silva, C.G. Superior operational stability of immobilized L-asparaginase over surface-modified carbon nanotubes. Sci Rep. 2021; 11(1), 21529.

[4] Orhan, H., Aktaş Uygun, D. Immobilization of L-Asparaginase on Magnetic Nanoparticles for Cancer Treatment. Appl Biochem Biotechnol 2020; 191, 1432–1443.

[5] Dik, G., Ulu, A., Inan, O. O., Atalay, S., Ateş, B. A Positive Effect of Magnetic Field on the Catalytic Activity of Immobilized L-Asparaginase: Evaluation of its Feasibility. Catal Lett 2023; 153, 1250–1264.

[6] Alharthi, F., Althagafi, H.A., Jafri, I., Oyouni, A.A.A., Althaqafi, M.M., Al-Hazmi, N.E., Al Hijab, L.Y.A., Naguib, D.M. Enhancing the Stability and Anticancer Activity of Escherichia coli Asparaginase Through Nanoparticle Immobilization: A Biotechnological Perspective on Nano Chitosan. Polymers 2024; 16, 3260.

[7] Monajati, M., Ariafar, N., Abedi, M., Borandeh, S., Tamaddon, A.M. Immobilization of L-Asparaginase on biofunctionalized magnetic graphene oxide nanocomposite: A promising approach for Enhanced Stability and reusability, Heliyon 2024; 10, Issue 21, e40072.

[8] Ulu, A., Noma, S.A.A., Koytepe, S., Ates, B. Magnetic Fe3O4@MCM-41 core–shell nanoparticles functionalized with thiol silane for efficient l-asparaginase immobilization, Artif Cells Nanomed Biotechnol 2018; 46, 1035-1045.

[9] Noma, S.A.A., Acet, Ö., Ulu, A., Önal, B., Odabaşı, M., Ateş, B. l-asparaginase immobilized p(HEMA-GMA) cryogels: A recent study for biochemical, thermodynamic and kinetic parameters, Polym. Test. 2021; 93, 106980.

[10] Monajati, M., Tamaddon, A.M., Abolmaali, S.S., Yousefi, G., Borandeh, S., Dinarvand, R. Enhanced L-asparaginase stability through immobilization in supramolecular nanogels of PEG-grafted poly HPMA with bis(α-cyclodextrin). Biochem. Eng. J. 2022; 191, 108802.

[11] Chahardahcherik, M., Ashrafi, M., Yousefi, G., Aminlari, M. Effect of chemical modification with carboxymethyl dextran on kinetic and structural properties of L-asparaginase, Anal. Biochem. 2020; 591, 113537.

[12] Barros, R.A.M., Cristóvão, R.O., Carneiro, I.G., Barros, M.A., Pereira, M.M., Carabineiro, S.A.C., Freire, M.G., Faria, J.L., Santos-Ebinuma, V.C., Tavares, A.P.M., Silva, C.G. Improved L-Asparaginase Properties and Reusability by Immobilization onto Functionalized Carbon Xerogels. ChemPlusChem. 2024; 89(9), e202400025.

[13] Monajati, M., Tamaddon, A.M., Abolmaali, S.S., Yousefi, G., Javanmardi, S., Borandeh, S., Heidari, R., Azarpira, N., Dinarvand, R. L-asparaginase immobilization in supramolecular nanogels of PEG-grafted poly HPMA and bis(α-cyclodextrin) to enhance pharmacokinetics and lower enzyme antigenicity. Colloids Surf B Biointerfaces. 2023; 225, 113234.

[14] Evli, S., Öndeş, B., Uygun, M., Uygun, D.A. Asparaginase immobilized, magnetically guided, and bubble-propelled micromotors, Process Biochem. 2021; 108, 103-109.

[15] de Melo, D.W., Fernandez-Lafuente, R., Rodrigues, R.C. Enhancing biotechnological applications of l-asparaginase: Immobilization on amino-epoxy-agarose for improved catalytic efficiency and stability, Biocatal Agric Biotechnol 2023; 52, 102821.

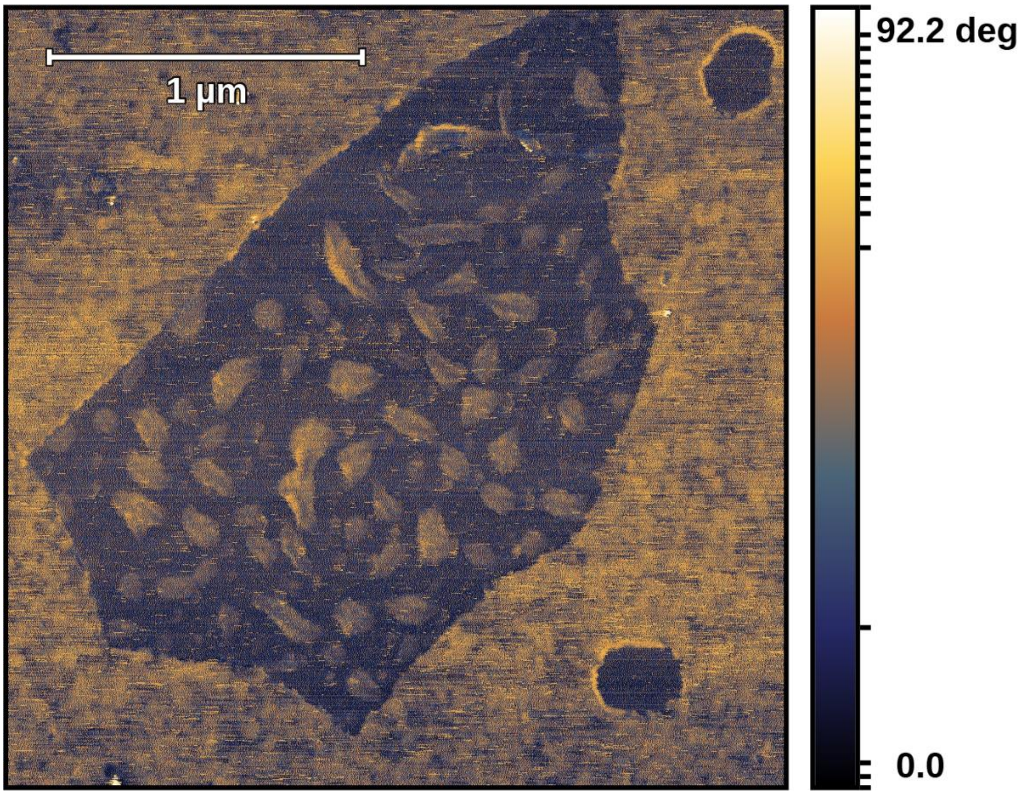

Figure S1. AFM phase image of L-ASNase on nGO sample.

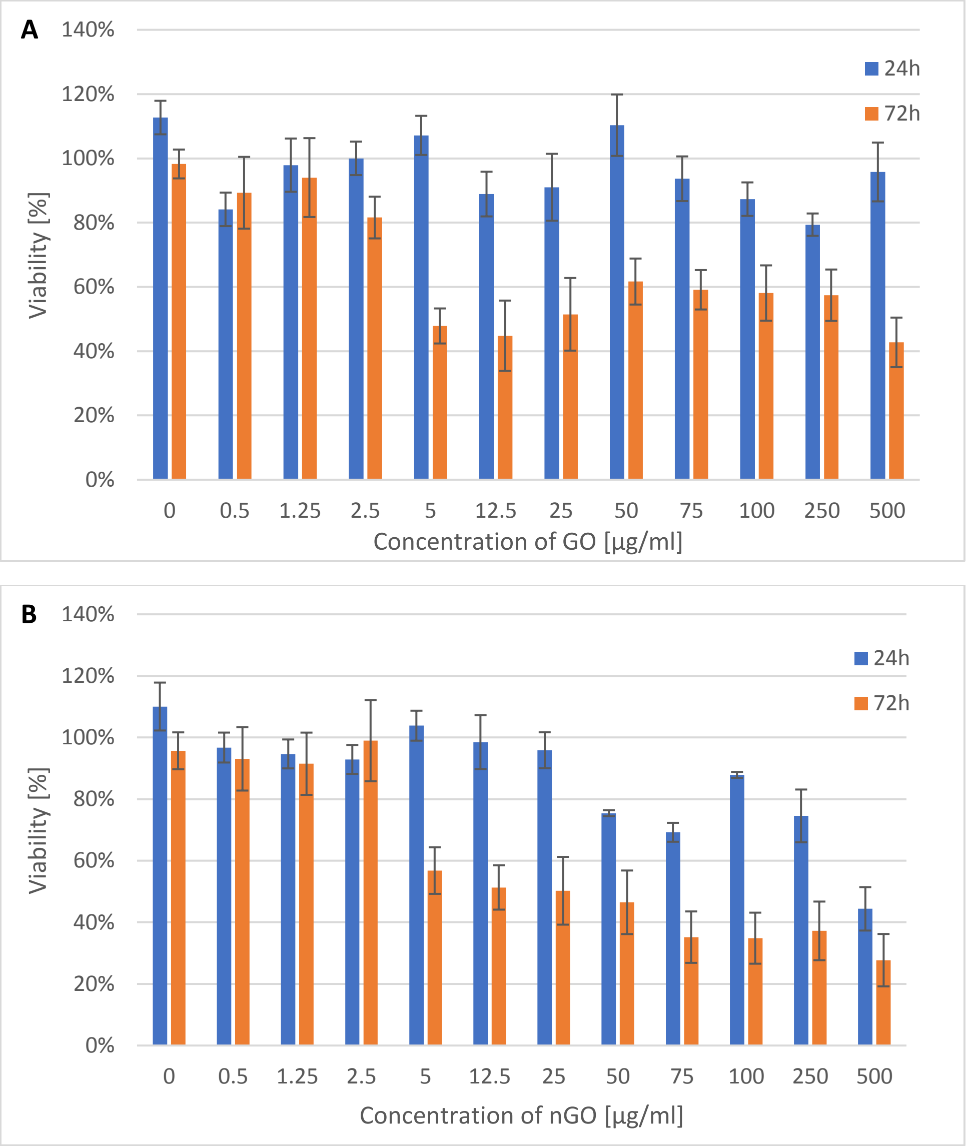

Figure S2. Concentration-dependent viability of HUVECs treated with GO (A) and nGO (B), and assayed with MTT test. The values are presented as mean ± SD (for n=4).

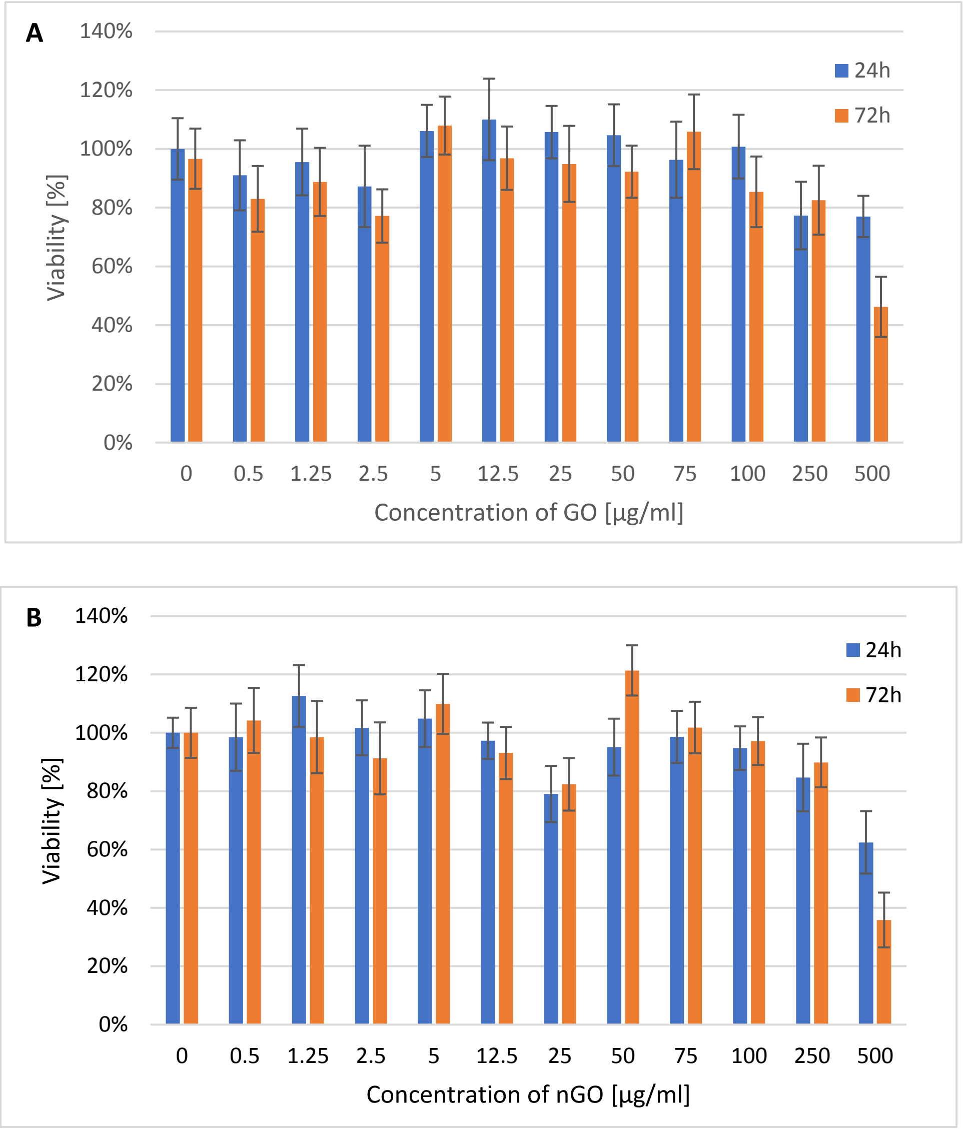

Figure S3. Concentration-dependent viability of HUVECs treated with GO (A) and nGO (B) and assayed with Neutral Red Uptake (NRU) test. The values are presented as mean ± SD (for n=4).

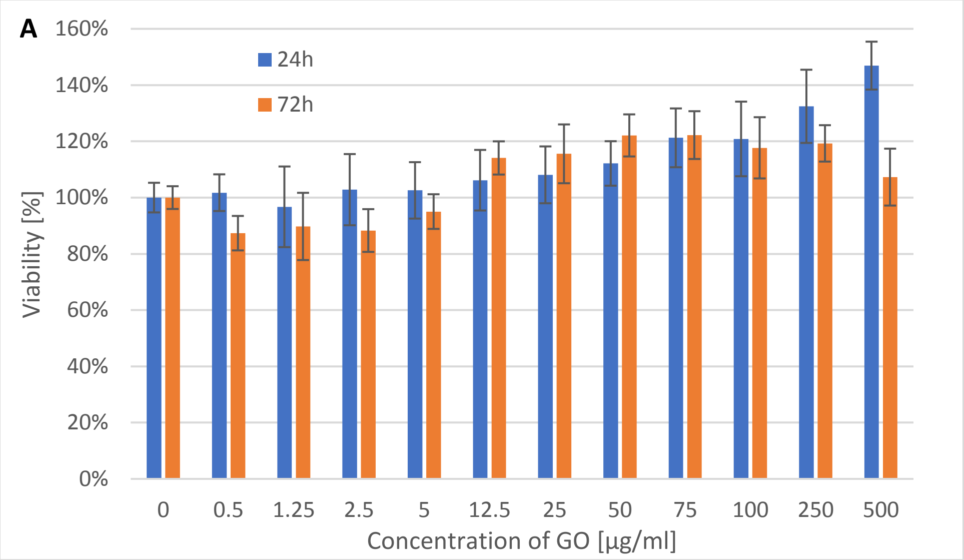

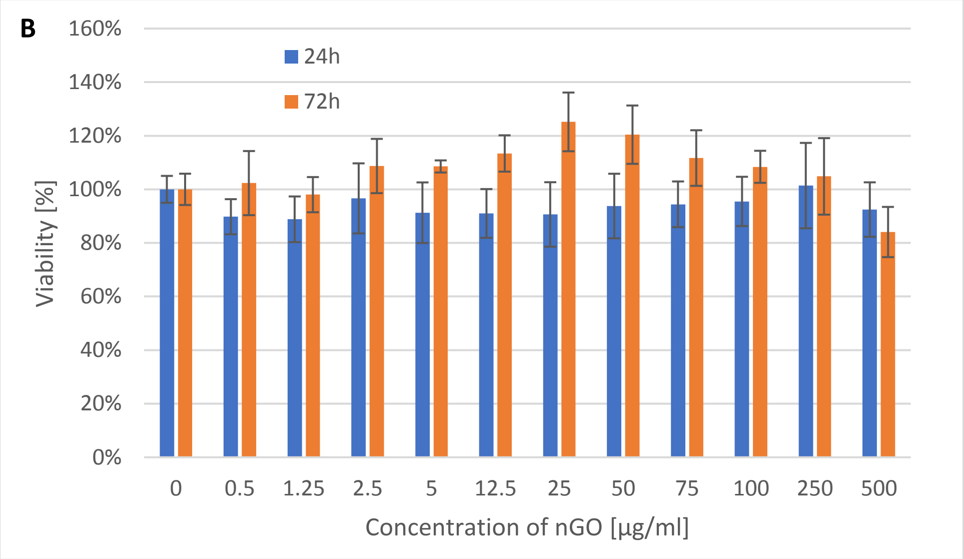

Figure S4. Concentration-dependent viability of K562 cells treated with GO (A) and nGO (B), and assayed with MTT test. The values are presented as mean ± SD (for n=4).

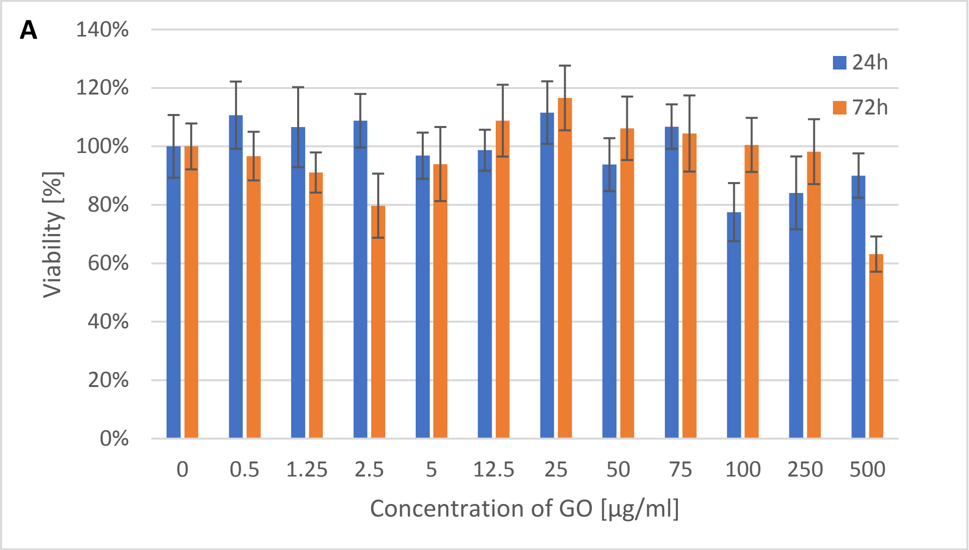

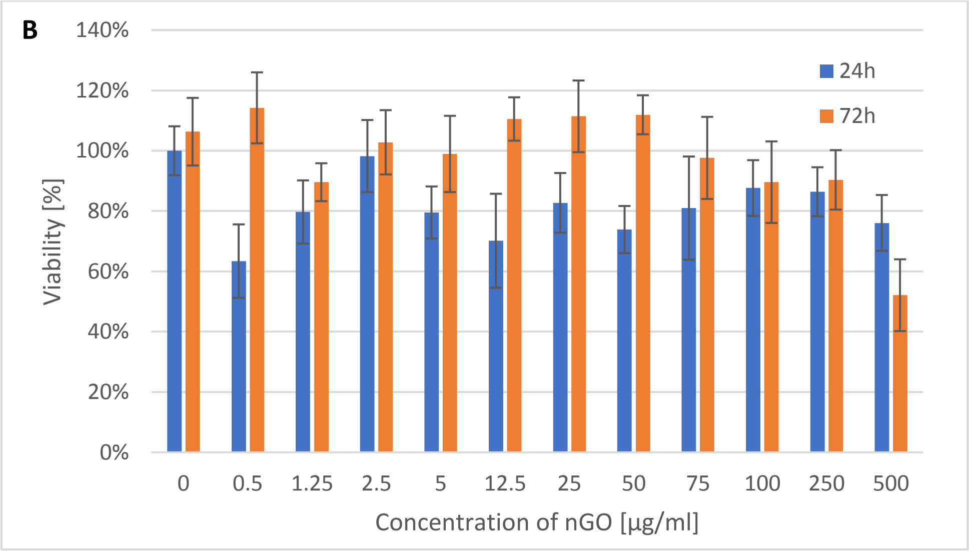

Figure S5. Concentration-dependent viability of K562 treated with GO (A) and nGO (B), and assayed with Neutral Red Uptake (NRU) test. The values are presented as mean ± SD (for n=4).

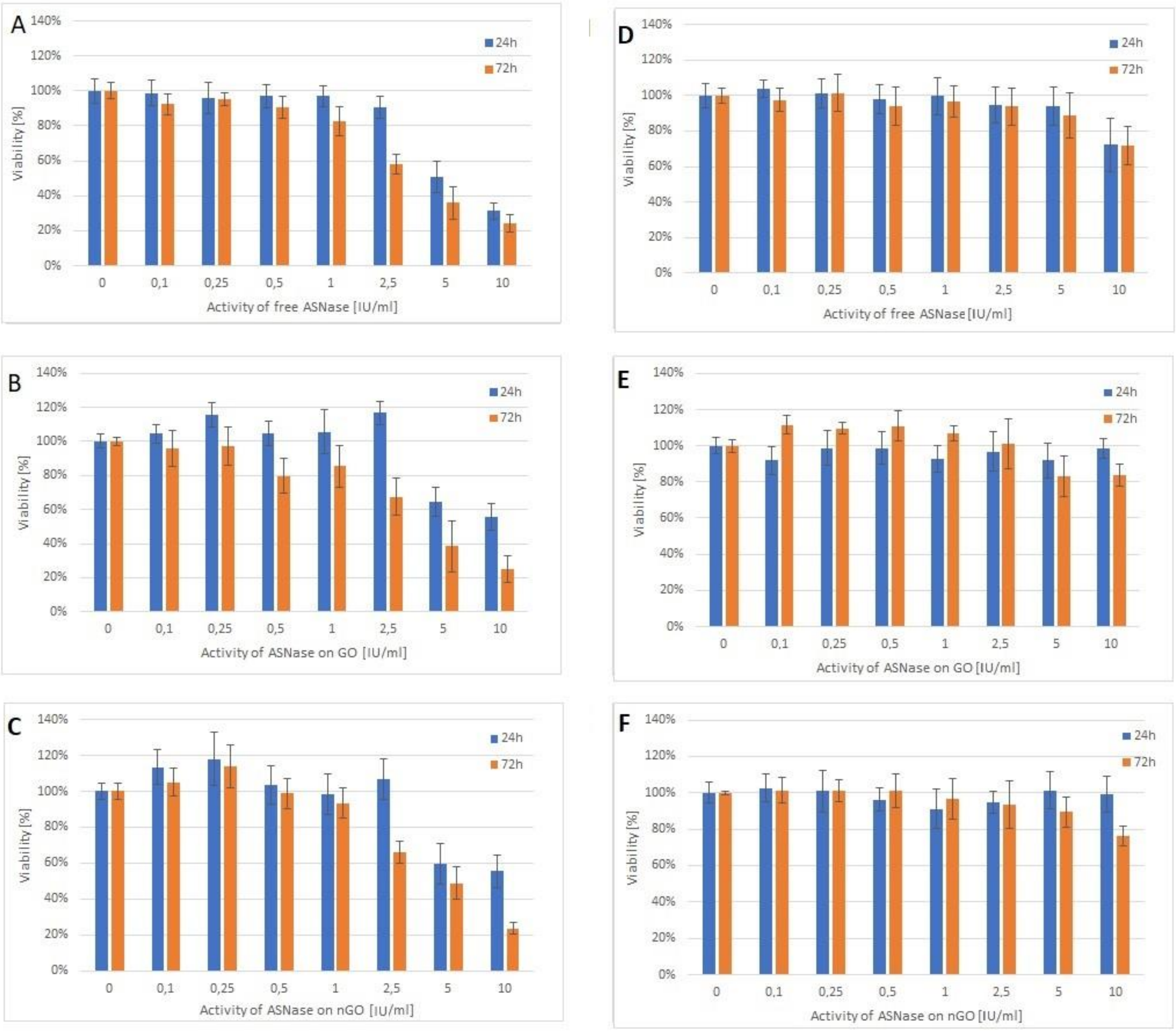

Figure S6. A, B, C – MTT viability assessment of HUVEC cells, D, E, F – MTT viability assessment of K562 cells in relation to different L-ASNase activities from 0.1 to 10 IU/mL. In case of immobilized enzyme, these activities correspond with 0.5, 1.25, 2.5, 5.0, 12.5, 25 and 50 µg/mL of support, respectively. The values are presented as mean ± SD (for n=4).

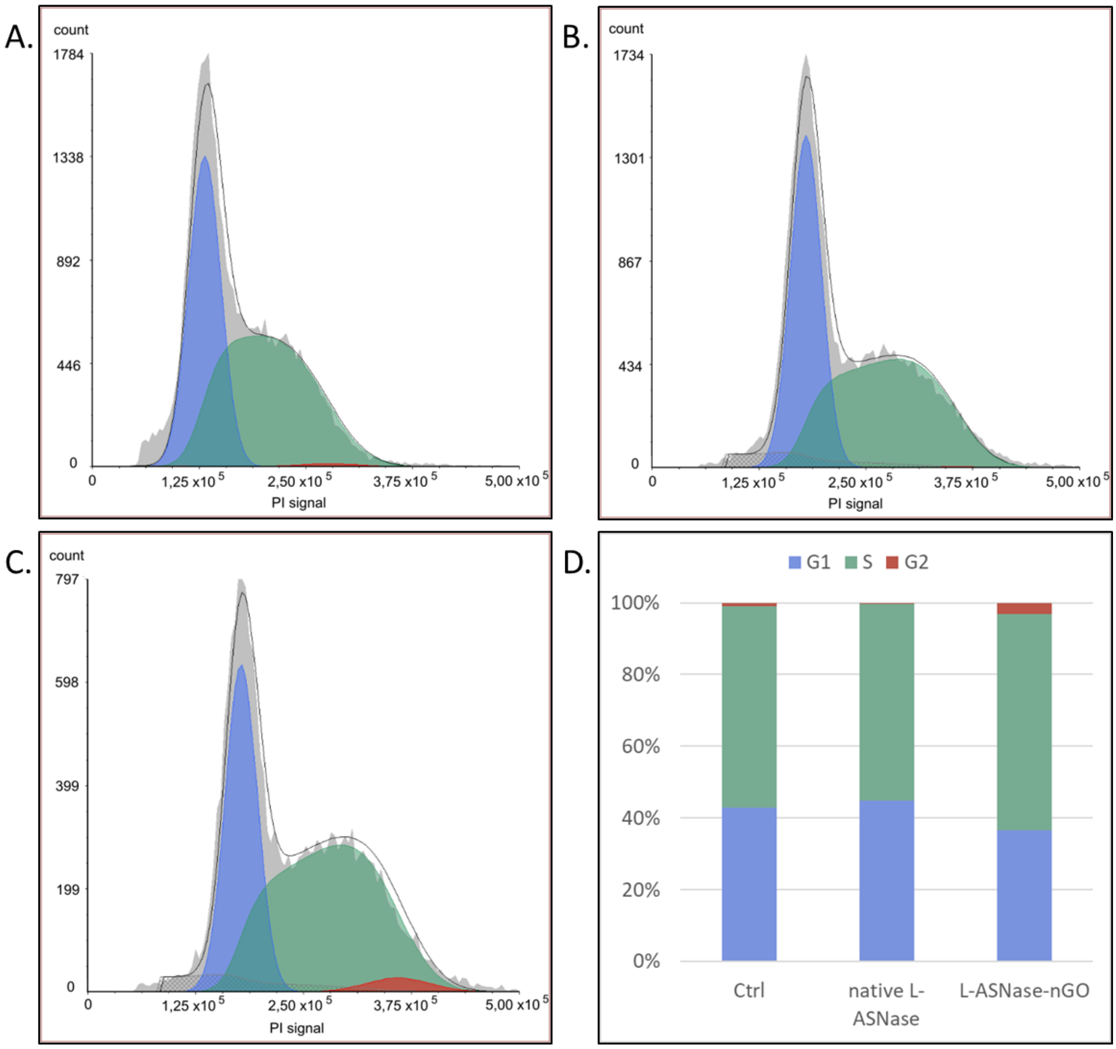

Figure S7. Cell cycle analysis through flow cytometry: A - control K562 cells, B - K562 cells treated with native L-ASNase, C – K562 cells treated with L-ASNase on nGO, D – percentage of cells in different phases of cell cycle after 72h-treatment.
